# Validation of the *qi blood yin yang deficiency* questionnaire on chronic fatigue

**DOI:** 10.1186/s13020-016-0092-y

**Published:** 2016-05-02

**Authors:** Jihye Kim, Boncho Ku, Keun Ho Kim

**Affiliations:** KM Fundamental Research Division, Korea Institute of Oriental Medicine, 1672 Yuseongdae-ro, Yuseong-gu, Daejeon, 34054 Republic of Korea

**Keywords:** Pattern identification, Deficiency pattern, Questionnaire, *Qi blood yin yang deficiencies*, Chronic fatigue

## Abstract

**Background:**

Chronic fatigue (CF) reflects an imbalance of inter-organ functions or of the four essential physiological components *qi*, *blood* (*xue*), *yin*, and *yang*. CF can be subdivided into different patterns. However, there are no diagnostic methods for CF. This study aimed to clinically validate a pattern identification method by identifying correlations between CF and responses to the *qi blood yin yang deficiency* questionnaire (QBYY-Q).

**Methods:**

Participants were recruited between May and June 2014 through the Kyung Hee University Korean Medicine hospital website and via posters and comprised 129 CF patients diagnosed with the United States Centers for Disease Control and Prevention (1994) criteria. Participants who had organic diseases that explained the CF were excluded. A total of 159 participants were asked to complete the QBYY-Q, the fatigue severity scale, and the Chalder fatigue scale. The latter two questionnaires were used to assess convergent validity with the QBYY-Q. Among the 129 CF participants, 70 and 59 had chronic fatigue syndrome and idiopathic chronic fatigue, respectively. Two Korean medical doctors independently assessed participants’ *qi*, *blood*, *yin*, and *yang deficiency* patterns using QBYY deficiency pattern identification guidelines. Based on the results of a preliminary study of the QBYY-Q, we selected 32 reliable items for symptoms corresponding to each *deficiency* pattern. The items were used to estimate internal consistency and construct validity. Multinomial logistic regression analysis was performed for scores on each *deficiency* pattern.

**Results:**

The data were means and standard deviations or numbers of participants and proportions for continuous and categorical variables, respectively. A statistical significance level of *P* < 0.05 was assumed. The QBYY-Q showed satisfactory internal consistency. Explanatory factor analysis extracted two factors for each *deficiency* pattern. The percentages of explained variance for *qi*, *blood*, *yin*, and *yang deficiency* were 45.1, 58.0, 52.2, and 63.4 %, respectively. Each QBYY-Q deficiency score was positively associated with each corresponding *deficiency* pattern. *Qi deficiency* was used as a reference category. Odds ratios of *blood*, *yin*, and *yang deficiency* were 10.97, 10.69, and 14.64, respectively.

**Conclusion:**

The QBYY-Q was suitable for estimating the influences of *qi*, *blood*, *yin*, and *yang deficiencies* in CF.

*Trial registration* This trial was registered with the Korean Clinical Trial Register (KCT0001199)

**Electronic supplementary material:**

The online version of this article (doi:10.1186/s13020-016-0092-y) contains supplementary material, which is available to authorized users.

## Background

Fatigue is a state of subjective tiredness and can be subdivided into prolonged fatigue and chronic fatigue (CF). CF is a condition of subjective tiredness and is reported by nearly 10 % of the global population [1]; it can be categorized as either explained CF or unexplained CF. Unexplained CF is further subdivided into chronic fatigue syndrome (CFS) and idiopathic chronic fatigue (ICF) [2]. CFS is characterized by severe disabling fatigue and a combination of four additional symptoms that may include impairments in cognitive or neurological function, sleep dysfunction, musculoskeletal pain, and endocrine or immune dysfunction (Table 1). Additionally, alternative medical and psychiatric causes must have been excluded, and the condition must have persisted for at least 6 months. ICF meets all the criteria for CFS except for the four additional symptoms.

**Table 1:** Diagnosis CDC-1994 criteria

Chronic fatigue syndrome symptoms
A. Major criteria (both criteria required)
1. Severe fatigue >6 months
a. Persistent or relapsing in course
b. Does not resolve with bed rest
c. Significant reduction in average daily activity (below 50 % of prior activity)
2. Other causes excluded
a. See fatigue causes
B. Minor criteria (4 or more present for 6 months)
1. Headache
a. New type, severity or pattern
b. Typically non-focal headache
2. Migratory polyarthralgias
a. Non-inflammatory (no swelling or erythema)
3. Myalgias
4. Postexertional malaise or fatigue
a. Duration >24 h
5. Short-term memory or concentration impaired
6. Pharyngitis
7. Tender cervical or axillary adenopathy
8. Non-restorative sleep

**Table 2:** Demographic characteristics of participants with CF (CFS and ICF) and controls

	Chronic fatigue		Controls (n = 30)	*P* value
	CFS (n = 6 5)	ICF (n = 56)		
Age (years)	29.7 ± 4.9	27.4 ± 5.2	26.0 ± 3.7	0.001
Sex (female)	42 (64.6)	20 (35.7)	15 (50.0)	0.007
BMI (kg/m^2^)	22.8 ± 3.5	22.3 ± 3.0	21.8 ± 3.1	0.746
SBP (mm Hg)	116.3 ± 11.0	116.8 ± 10.2	119.2 ± 12.7	0.471
DBP (mm Hg)	69.5 ± 11.1	71.8 ± 9.0	71.2 ± 12.5	0.471
Pulse rate (beats/min)	71.4 ± 8.9	70.0 ± 6.3	72.0 ± 9.5	0.482
Body temperature (°C)	36.4 ± 0.2	36.4 ± 0.2	36.4 ± 0.2	0.601
Smoking	18 (27.7)	13 (23.2)	4 (13.3)	0.305
Regular exercise	20 (30.8)	27 (48.2)	12 (40)	0.145
Regular diet	36 (55.4)	33 (58.9)	19 (63.3)	0.76
Pattern identification				
QD	16 (24.6)	13 (23.2)	14 (46.7)	0.129
BD	13 (20)	18 (32.1)	6 (20)	
YnD	16 (24.6)	14 (25)	6 (20)	
YgD	20 (30.8)	11 (19.6)	4 (13.3)	

The data are represented as the means ± SDs or as numbers of participants and proportions; N (%) for continuous and categorical variables, respectively. One-way ANOVA was performed for continuous variables and Pearson’s χ^2^ test for categorical variables

*CFS* chronic fatigue syndrome, *ICF* idiopathic chronic fatigue, *SBP* systolic blood pressure, *DBP* diastolic blood pressure, *QD* *qi deficiency*, *BD* *blood deficiency*, *YnD yin deficiency*, *YgD* *yang deficiency*

**Table 3:** Agreement results in the KMD diagnoses (n = 157)

KMD diagnoses		KMD #1			
		QD	BD	YnD	YgD
KMD #2	QD	43	0	0	1
	BD	1	37	1	0
	YnD	1	2	36	0
	YgD	0	0	0	35

*KMD* Korean medical doctor, *QD* *qi* *deficiency,* *BD* *blood deficiency,* *YnD* *yin deficiency,* *YgD* *yang deficiency*

Studies of CFS and ICF indicate prevalence rates of approximately 10 and 1 % of the general population, respectively [3]. The estimated prevalence of CF in the Republic of Korea is between 0.6 and 2 % of the general adult population [4]. Several hypotheses have been proposed for the pathogenesis of CF, including oxidative stress, hypothalamic–pituitary–adrenal axis abnormalities, and immune dysfunction [5–7]. Western medicine offers no objective diagnostic methods or effective therapies for CF and the recommended drugs have many side effects [8–11]. CFS and ICF patients tend to be interested in complementary and alternative medicine treatments [9, 12, 13].

An individual’s constitution and current health status can be conceptualized in terms of the balance between the *qi* and *blood* (*xue*) conditions of the internal organ functions and of the co-existing *yin* and *yang* (which constitute the physical form and functioning of the human body and its deficient and excessive energetic qualities) [9, 14, 15]. Treatments such as acupuncture, moxibustion, herbal medicine, and qigong restore the balance of the four essential components of the human body: *qi*, *blood*, *yin*, and *yang*.

Unexplained CF can be classified as either a *deficiency* syndrome or an *excess* syndrome; these syndromes refer to the *deficiency* or excess of *qi*, *blood*, *yin*, and *yang* [16, 17]. Unexplained CF is usually considered a *deficiency* pattern representing an imbalance of the four essential components; most cases are treated by invigorating *qi* and *yang* and nourishing *blood* and *yin* [9, 18, 19]. CF treatments are based on individual patterns of body constitution or pattern identification (PI); most patients are diagnosed according to four patterns of body constitution that involve the five major organs: the heart, liver, spleen, lungs, and kidney. These patterns are *qi deficiency* (QD, i.e., decreases in visceral functions and body resistance), *blood deficiency* (BD, i.e., failure to nourish the organs, tissues, and meridians), *yin deficiency* and *yang deficiency* (YnD and YgD, i.e., failure to maintain normal function of internal organs) [9, 11, 18].

This study aimed to clinically validate a PI diagnostic method by testing for correlations between CF and responses to the *qi bloodyin yang deficiency* questionnaire (QBYY-Q).

## Methods

### Ethics

The study protocol was reviewed and approved by the Institutional Review Board of the Cheonan Oriental Hospital at Daejeon University (authorization number: 2014-A01-02) and was registered with the clinical research information service (registration number: KCT0001199) (Additional file 1).

### Study design

**Fig. 1:**
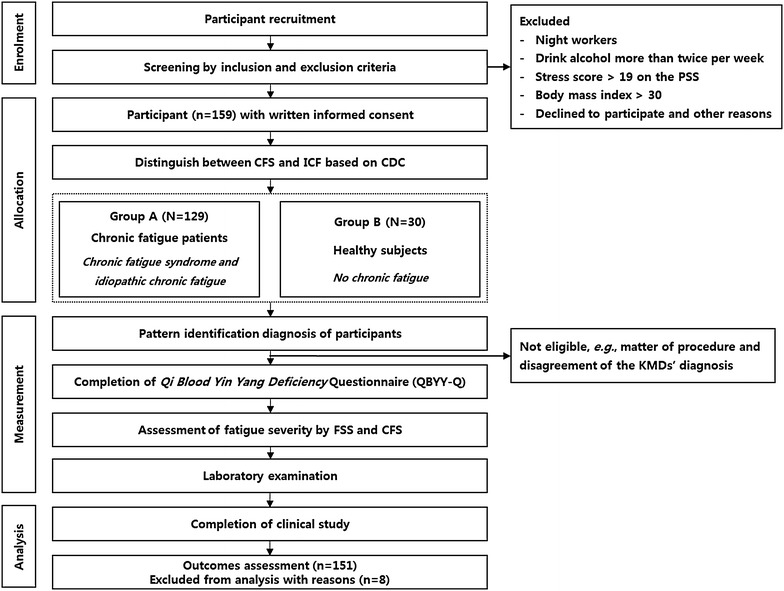
Flow diagram of the procedures of the clinical study

This study was designed as a single-center, cross-sectional, case-controlled study, and it was performed at the Cheonan Oriental Hospital of Daejeon University in Cheonan, South Korea from May to June 2014. The procedures of this study were depicted in Fig. 1.

### Participants

Participants were recruited by advertising on hospital websites and posters displayed in hospitals and local universities, Seoul, Republic of Korea from May to June 2014. They went through telephone pre-screening by the clinical study coordinator to ensure that CF patients met the inclusion criteria of “males and females aged 19–39’’ and “the presence of CF that is continuous and repetitive for more than 6 months”. Then, participants visited the clinical study center and go through a more in-depth screening process to determine whether they satisfied the inclusion criteria.

Participants who worked at night or drank alcohol more than twice per week were excluded. We excluded the participants who had organic diseases for explained CF, such as acute or chronic liver disease (e.g., hepatitis, and liver cirrhosis); anemia; tuberculosis; chronic lung disease; cardiovascular disease (e.g., heart failure, hypertension); endocrine/metabolic disease (e.g., diabetes, thyroid gland disease, and severe obesity); autoimmune disease (e.g., rheumatoid arthritis, systemic lupus erythematosus, and multiple sclerosis); malignant tumors; or infectious disease were excluded. In addition, CF patients with psycho-social diseases, such as depression, anxiety neurosis, recent severe stress, schizophrenia, alcoholism, or an eating disorder (e.g., anorexia nervosa, bulimia nervosa), were excluded [12, 13]. Cut-offs of BMI (≥30 kg/m^2^) have shown good specificity in diagnosing severe obesity, which is defined as BMI ≥30 kg/m^2^ based on measured weight and height and is a type of metabolic disease [20, 21]. The participants were informed of the entire process of the clinical study and were asked to sign informed consent forms (Additional file 2).

The final participants included 129 patients with unexplained CF and 30 controls. The enrolled patients mainly suffered from fatigue lasting more than 6 months. The CDC-1994 criteria for CFS [22] were used to distinguish between CFS and ICF, using the cutoff of 4 among the 8 symptoms in the minor criteria, respectively (Table 1). CFS patients complained of major criteria symptoms and more than four of the minor criteria symptoms, and ICF patients complained of major criteria symptoms and less than four of the minor criteria symptoms. If a subject complained of fatigue lasting more than 6 months, headache and pharyngitis, the subject was diagnosed as ICF. Among the attending 129 CF patients, 70 and 59 were found to have CFS and ICF, respectively.

The control (30 healthy participants) came to the same clinic who worked at night and drank alcohol were excluded. The control group consisted of participants who satisfied the following criteria: (1) not meeting the CFS and ICF criteria from CDC-1994; (2) absence of organopathy; and (3) normal results of laboratory tests and radiological examinations.

Of the 159 participants, two participants were excluded due to noncompliance with the process described in the protocol; therefore, 157 participants were included in this study.

### Pattern diagnosis

There is no “gold standard” for pattern diagnosis. Our previous study demonstrated discrepancies in the diagnoses of QD, BD, YnD, and YgD among Korean medical doctors (KMDs) [23]. To increase the internal consistency of KMD expert pattern diagnosis, we created PI guidelines for QD, BD, YnD, and YgD with PI experts, using data from previous studies [9, 24] and the standard Korean medical literature [22] 4 months before the study commenced. The developed PI guidelines were validated after consultation with experts. Based on the PI guidelines, two KMDs with 4 years of clinical experience independently diagnosed the participants’ *deficiency* status; the KMDs were blinded to the results of the diagnoses. Participants for whom the KMD diagnoses disagreed were excluded from the analysis.

## Measurements

### QBYY-Q scales

In our previous studies, items related to QD, YnD, YgD, and BD were systematically reviewed [9, 25–27], and a QBYY-Q constructed by a panel of experts was selected for use in a pilot study. This QBYY-Q had been established by an expert panel of 27 KMDs in a previous study [28]. A pilot study was performed to determine the reliability and validity of the QBYY-Q for 100 participants with CF [23]. After these participants completed the QBYY-Q, the KMDs divided the patients into four groups: QD, BD, YnD, and YgD. After 3 weeks, the 100 participants were retested in the same way. The results from our pilot study showed that Cronbach’s α coefficient was 0.916 for internal consistency of the QBYY-Q. Construct validity analyzed using exploratory factor analysis (EFA) produced four factors with eigenvalues greater than 1.0. Factors 1, 2, 3, and 4 were identified as QD, YnD, YgD, and BD, respectively. However, four items, “menstrual cycle slowdown,” “decreased masculine energy,” “damp and cold penis,” and “decreased sexual desire,” produced an item-total correlation of less than 0.5. Because the results indicated that the four items were not equivalent to the others, these items were excluded from the validity tests. Test–retest reliability was high (intraclass correlation coefficient = 0.699) [23]. Consequently, the final version of the QBYY-Q included 32 sign and symptom entries consisting of nine QD, eight BD, nine YnD, and six YgD pattern items. The order of the 32 QBYY-Q pattern items was reversed to reduce bias (Additional file 3). The QD pattern included two signs, enervation and shortage of *qi* or faint breathing, and the BD pattern included three signs, pale complexion, sweating during the day, and palpitations (a subjective sensation of rapid and forceful beating of the heart). The YnD pattern was divided into following two signs: (1) dry skin and mouth; and (2) heat vexation and fever accompanied by uneasiness or restlessness. The YgD pattern was divided into two signs, cold in the extremities up to the knees and elbows and diarrhea. The severity of each item was graded using the following 4-point scale: 1 = strongly disagree, 2 = disagree, 3 = agree, and 4 = strongly agree. The scores for each item were totaled, and QD-Q, BD-Q, YnD-Q, and YgD-Q scores calculated.

### Fatigue severity scale (FSS)

The FSS is a self-rating questionnaire comprising nine items that are scored between 1 (completely disagree) and 7 (completely agree). The items assess the extent of fatigue symptoms and their impact on patient functioning (including motivation; exercise; physical functioning; ability to perform duties; and interference with work, family, or social life) [29]. Example items on the questionnaire are “exercise brings on my fatigue” and “my fatigue is very debilitating”; higher item scores indicate a greater degree of fatigue.

### Chalder fatigue scale–Korean version (CFS-K)

The CFS is a self-reported questionnaire that measures fatigue intensity on a 4-point scale (0 = less than usual to 3 = much more than usual) [30]. Symptoms assessed by the scale are divided into two subcategories: seven items related to physical symptoms, and four items related to mental fatigue. Although the internal consistency of the CFS-K has not been fully reported, many studies using versions of the CFS in different languages have shown strong internal consistency for both the physical and mental symptoms of fatigue. Likert scoring with weights was used to score the magnitude of fatigue [30].

### Statistical analysis

The data were represented as means ± standard deviations (SDs) or as numbers of participants and proportions for continuous and categorical variables, respectively. All statistical analyses were performed using R software (The R Foundation), version 3.1.1, on a Windows 7 platform. R software is a free software environment for statistical computing and graphics [31]. The level of significance was set at *P* < 0.05 for all analyses. Inter-rater agreement for the two KMDs’ diagnoses of QD, BD, YnD, and YgD was evaluated using Cohen’s κ coefficient. Differences between CF patients (CFS and ICF) and the non-CF group were tested using a one-way ANOVA for continuous variables and Pearson’s Chi square test for categorical variables. The Keiser–Meyer–Olkin (KMO) test was used to test sample adequacy (considered acceptable if the KMO constant is >0.60) [32]. The KMO was used to check the matrix of correlations for the applicability of factorial analysis, and then a principal axis factoring extraction method was applied. Construct validity for each *deficiency* scale on the questionnaire was estimated by performing EFA with a combination of minimum residual extraction and promax rotation. For each *deficiency* scale, conceptual factors were identified by examining the factor loadings. The internal consistency of each *deficiency* scale was also tested using Cronbach’s α coefficient. Convergent validity of *deficiency* scores derived from the four QBYY-Q scales and the total scores on the CFS-K and FSS was assessed using Spearman’s correlation coefficient. The mean differences in *deficiency* scores between the CF and non-CF groups were examined using a one-way ANOVA test. In addition, binary logistic regression analysis was used to estimate the odds ratios (ORs) of CF (CFS + ICF versus no CF) in the KMD diagnoses of QD, BD, YnD, and YgD to confirm the associations between CF and PI. Both CF and PI could have been influenced by other confounding factors; therefore, age and sex were included to estimate the adjusted ORs for CF. Discriminative validity was determined using the ORs of the four *deficiency* scale scores derived from a multinomial logistic regression model.

## Results

### Demographic characteristics

Because of the absence of a gold standard for pattern diagnosis, six of the 157 participants showed discrepancies in pattern diagnoses and were excluded to increase the internal consistency. Therefore, 151 participants were included in the analysis. Table 2 shows the demographic characteristics of the 65 participants with CFS, the 56 participants with ICF, and the 30 control subjects in the non-CF group. For most of the characteristics, there were no significant differences among the CF groups (CFS and ICF) and the control group. However, the age and sex distributions showed significant differences among the three groups. The means and SDs for age were 29.7 ± 4.9 years for participants with CFS, 27.4 ± 5.2 years for participants with ICF, and 26.0 ± 3.7 years for the control group (*F* = 7.09, *P* = 0.001). The proportions of women in the CFS, ICF, and control groups were 64.6, 35.7, and 50.0 %, respectively (χ^2^ = 10.07, *P* = 0.007).

### KMD inter-rater agreement

Cohen’s κ coefficient was 0.95 (Z = 20.69, *P* < 0.001), which indicated that the agreement between the two KMDs was sufficiently strong. The two KMDs differed in their PI diagnoses for six (3.8 %) of the 157 participants (Table 3).

### Internal consistency and construct validity of the QBYY-Q

The internal consistency and construct validity of each of the QBYY-Q scales were assessed by merging the current study and pilot study data (totaling 252 cases). The Cronbach’s α coefficients of the nine QD items (QD-Q), the eight BD items (BD-Q), the nine YnD items (YnD-Q), and the six YgD items (YgD-Q) were 0.816, 0.826, 0.807, and 0.717, respectively.

KMO tests of sphericity confirmed that each QBYY-Q scale contained an eligible number of items and a sufficient sample size (QD-Q = 0.856, BD-Q = 0.833, YnD-Q = 0.807, YgD-Q = 0.702). Two factors were extracted for each QBYY-Q scale following EFA with minimum residual extraction and promax rotation. The EFA results for each QBYY-Q scale are shown in Table 4.

**Table 4:** Extracted factor loadings of items for each QBYY-Q and the variance explained by two factors

PI	Item	Sub-scale	QD-Q		BD-Q		YnD-Q		YgD-Q	
			FL1	FL2	FL1	FL2	FL1	FL2	FL1	FL2
QD	QD-Q 01	Fatigue	*0.821*	−0.054						
	QD-Q 02		*0.798*	−0.087						
	QD-Q 05		*0.824*	−0.033						
	QD-Q 06		*0.444*	0.043						
	QD-Q 07		*0.367*	−0.107						
	QD-Q 09		*0.287*	0.160						
	QD-Q 03	Weakened voice	−0.147	*1.098*						
	QD-Q 04		0.231	*0.330*						
	QD-Q 08		0.171	*0.433*						
	% of variance explained		27.90	17.20						
BD	BD-Q 01	Heart pounding			*0.831*	−0.081				
	BD-Q 02				*0.865*	−0.182				
	BD-Q 03				*0.537*	0.098				
	BD-Q 08				*0.537*	0.016				
	BD-Q 04	Pale and dry body			0.112	*0.499*				
	BD-Q 05				0.090	*0.617*				
	BD-Q 06				−0.222	*0.977*				
	BD-Q 07				0.308	*0.313*				
	% of variance explained				45.30	12.70				
YnD	YnD-Q 02	Rough and dry body					*0.508*	0.145		
	YnD-Q 05						*0.712*	−0.120		
	YnD-Q 06						*0.770*	−0.102		
	YnD-Q 07						*0.689*	−0.033		
	YnD-Q 01	Body steaming					0.243	*0.267*		
	YnD-Q 03						−0.042	*0.741*		
	YnD-Q 04						0.216	*0.448*		
	YnD-Q 08						−0.154	*0.848*		
	YnD-Q 09						0.054	*0.388*		
	% of variance explained						39.60	12.60		
YgD	YgD-Q 01	Preference for warm things							*0.821*	−0.120
	YgD-Q 02								*0.560*	0.012
	YgD-Q 03								*0.692*	−0.072
	YgD-Q 04								*0.417*	0.225
	YgD-Q 05	Diarrhea							−0.146	*0.939*
	YgD-Q 06								0.052	*0.646*
	% of variance explained								41.50	21.90

The italic letters represents the largest loading between two factors

*PI* pattern identification, *QD* *qi deficiency*, *QD*-*Q* *qi deficiency* questionnaire, *BD* *blood deficiency*, *BD*-*Q* *blood deficiency* questionnaire, *YnD* *yin deficiency*, *YnD*-*Q* *yin deficiency* questionnaire, *YgD* *yang deficiency*, *YgD*-*Q* *yang deficiency* questionnaire, *FL* factor loading

### Convergent validity of the QBYY-Q

The convergent validity of each QBYY-Q scale was assessed with the FSS and CFS-K using pairwise Spearman’s correlation coefficients (Table 5). Total *deficiency* scores for the QD-Q, BD-Q, YnD-Q, and YgD-Q were calculated by averaging all the items corresponding to each *deficiency* scale. Subscores for each *deficiency* scale were also obtained by averaging the items relevant to each factor, which were identified from the EFA. The QD-Q score correlated significantly with both the FSS (*r* = 0.721, *P* < 0.001) and the CFS-K (*r* = 0.645, *P* < 0.001). The BD-Q score also correlated significantly with the FSS (*r* = 0.44, *P* < 0.001) and the CFS-K (*r* = 0.47, *P* < 0.001). The YnD-Q score correlated significantly with the FSS (*r* = 0.41, *P* < 0.001) and the CFS-K (*r* = 0.48, *P* < 0.001). The YgD-Q score also correlated significantly with the FSS (*r* = 0.40, *P* < 0.001) and the CFS-K (*r* = 0.29, *P* < 0.001). Most of the subscales for the *deficiency* scales were significantly correlated with both the CFS-K and the FSS, with correlations ranging from 0.295 to 0.704. However, YgD-S2 was not significantly correlated with either the CFS-K (*r* = 0.083, *P* = 0.314) or the FSS (*r* = 0.152, *P* = 0.063).

**Table 5:** Pairwise Spearman’s correlation coefficients of QBYY-Q scores and sub-scales of the QD-Q, BD-Q, YnD-Q and YgD-Q with the CFS-K and FSS

	CFS-K	*P* value	FSS	*P* value
QD-Q score	0.645	<0.001	0.721	<0.001
QD-Q S1: Fatigue	0.653	<0.001	0.704	<0.001
QD-Q S2: Weakened voice	0.419	<0.001	0.484	<0.001
BD-score	0.467	<0.001	0.441	<0.001
BD-Q S1: Heart pounding	0.298	<0.001	0.366	<0.001
BD-Q S2: Pale and dry body	0.481	<0.001	0.411	<0.001
YnD-score	0.485	<0.001	0.408	<0.001
YnD-Q S1: Rough and dry body	0.387	<0.001	0.252	0.0018
YnD-Q S2: Body steaming	0.411	<0.001	0.433	<0.001
YgD-score	0.291	<0.001	0.403	<0.001
YgD-Q S1: Preference for warm things	0.295	<0.001	0.384	<0.001
YgD-Q S2: Diarrhea	0.083	0.314	0.152	0.063

*CFS*-*K* Korean version of the Chalder fatigue scale, *FSS* fatigue severity scale, *QD*-*score* *qi deficiency* score, *BD*-*score* *blood deficiency* score, *YnD*-*score* *yin deficiency* score, *YgD*-*score* *yang deficiency* score

### Least square means of QBYY-Q scores with CF status

**Fig. 2:**
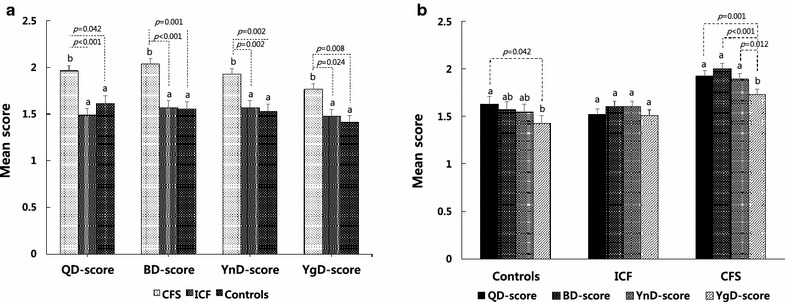
Least mean square plot for QBYY-Q scores. **a** Least mean squares of each QBYY-Q score according to the control, ICF, and CFS groups; **b** Least mean squares of QBYY-Q scores among the control, ICF, and CFS groups after adjustment for sex and age. *Dotted lines* denote the significant pairwise comparisons in which the *P* value was less than 0.05. Alphabetic notations (i.e., a, ab and b) above the standard *error bars* indicate the homogeneous groups resulting from Tukey’s HSD test for multiple comparisons

The least square means of each QBYY-Q scale score with CF status were acquired after multivariate adjustment for sex and age. Each marginal mean corresponding to a QBYY-Q score was estimated from the linear mixed model. The least square means of each QBYY-Q scale for the CF and the control groups are shown in Fig. 2a. The total scores for the QD-Q, BD-Q, YnD-Q, and YgD-Q for the CFS patients were 1.962, 2.038, 1.928, and 1.767, respectively, and those for the control group were 1.615, 1.554, 1.530, and 1.411, respectively. Patients with a diagnosis of CFS showed significantly higher QD, BD, and YnD, and YgD scores than those diagnosed with ICF (QD-Q score: *P* < 0.001; BD-Q score: *P* = 0.001, YnD-Q score: *P* = 0.002; YgD-Q score: *P* = 0.024) or those in the control group (QD-Q score: *P* = 0.042; BD-Q score: *P* = 0.001; YnD-Q score: *P* = 0.002; YgD-Q score: *P* = 0.008). The profiles of the mean QBYY-Q scores among the control, ICF, and CFS groups are shown in Fig. 2b. The mean difference between the QD score and YgD score was significant for both the control (*P* = 0.042) and the CFS group (*P* = 0.001). There were no significant differences among QBYY-Q scores for the ICF group.

### Association between PI and CF

To analyze the association between the KMD diagnosis and CF, the ICF and CFS groups were merged into one large CF group. Table 6 shows the crude and adjusted ORs for the association between the KMD PI diagnoses and CF, which were acquired through binomial logistic regression. The crude ORs for BD, YnD, and YgD were significantly higher than the OR for QD (2.49-, 2.41-, and 3.74-fold, respectively). The prevalence ORs of BD, YnD, and YgD in model 1 were 3.41-, 3.41-, and 4.68-fold higher, respectively, than the OR of QD. These results were statistically significant (*P* < 0.05).

**Table 6:** Crude and adjusted odds ratios of CF by PI

PI	Crude	Model 1
QD	Reference	Reference
BD	2.49 (0.88, 7.85)	3.41 (1.10, 11.87)*
YnD	2.41 (0.85, 7.61)	3.41 (1.13, 11.48)*
YgD	3.74 (1.19, 14.40)*	4.68 (1.35, 19.65)*

*Odds ratio* OR (95 % confidence interval), *qi deficiency* was used as a reference category, *Model 1* adjusted for sex and age*, QD* *qi deficiency*, *BD* *blood deficiency*, *YnD* *yin deficiency*, *YgD* *yang deficiency*, *PI* pattern identification

* <0.05, ** <0.01, *** <0.001

### Associations between PI and CFS and QBYY-Q scores

A multinomial logistic regression was used to examine associations between PI and QBYY-Q scores. The resulting values were ORs (95 % confidence intervals) with mean increments and are shown in Table 7. Using QD as a reference category for all PI scores, the BD, YnD, and YgD scale scores were positively associated with each corresponding *deficiency* pattern (adjusted ORs: BD-Q score = 5.877, YnD-Q score = 12.570, YgD-Q score = 13.558).

**Table 7:** Associations between PI and QBYY-Q scores

		QD	BD	YnD	YgD
Crude	QD-Q score	Reference	0.05 (0.01, 0.26) ***	0.17 (0.02, 0.61) **	0.07 (0.01, 0.44) *
	BD-Q score	Reference	10.97 (2.10, 57.19) **	1.25 (0.25, 6.32)	4.99 (0.90, 27.60)
	YnD-Q score	Reference	1.32 (0.21, 8.18)	10.69 (1.72, 66.42) *	0.66 (0.10, 4.51)
	YgD-Q score	Reference	0.64 (0.17, 2.32)	0.28 (0.07, 1.13)	14.64 (3.80, 56.38) ***
Model 1	QD-Q score	Reference	0.04 (0.01, 0.24) ***	0.12 (0.02, 0.66) *	0.05 (0.01, 0.40) **
	BD-Q score	Reference	5.88 (1.04, 33.12)	1.07 (0.20, 5.79)	2.39 (0.38, 14.18)
	YnD-Q score	Reference	2.12 (0.32, 14.19)	12.57 (1.90, 83.07)**	1.22 (0.16, 9.20)
	YgD-Q score	Reference	0.53 (0.14, 2.01)	0.27 (0.07, 1.10)	13.56 (3.40, 53.99) ***

*Odds ratio* OR (95 % confidence interval) obtained from multinomial logistic regression, *qi deficiency* was used as a reference category, *Model 1* adjusted for sex and age, *QD* *qi deficiency*, *QD*-*Q* *qi deficiency* questionnaire, *BD* *blood deficiency*, *BD*-*Q* *blood deficiency* questionnaire, *YnD* *yin deficiency*, *YnD*-*Q* *yin deficiency* questionnaire, *YgD* *yang deficiency*, *YgD*-*Q* *yang deficiency* questionnaire, *FL* factor loading

* <0.05, ** <0.01, *** <0.001

## Discussion

In this study, we aimed to clinically develop a PI questionnaire and to analyze the effectiveness of the four QBYY-Q scales as diagnostic tools for CF subtypes. We examined the correlations between CF and the QBYY-Q scales using KMD inter-rater agreement, the internal consistency and construct validity of the QBYY-Q, demographic characteristics, the convergent validity of the QBYY-Q, the least square means of the QBYY-Q scores with CF status, the associations between PIs and CF, and associations between PI and CF and QBYY-Q scores.

We compared the sociodemographic and clinical characteristics of CF patients with those of healthy subjects; however, it was difficult to delineate the clinical characteristics of CF in our study because of the small number of participants, i.e., 121 patients and 30 healthy subjects. In contrast to previous studies, the current study found no sex differences among groups, but it showed significant differences among groups for marital status and sex [33, 34].

Validation of the QBYY-Q and the Delphi methods were conducted using two different groups of clinicians, which strengthened the discriminant ability of the QBYY-Q [23, 28]. The revised QBYY-Q exhibited satisfactory internal consistency, with a Cronbach’s α of 0.900 for the overall signs and symptoms, and the internal consistency of each pattern was satisfactory (0.717–0.826). The strong internal consistency reliability for each pattern suggested that the pattern constructs were homogenous or that the signs and symptoms were appropriate measures of these *deficiency* syndrome constructs.

The two QD factors were identified as weakness and shortage of *qi* or faint breathing. The two BD factors were identified as pale complexion and palpitations, i.e., a subjective sensation of rapid and forceful beating of the heart. *Yin* and *yang deficiencies* were divided into two factors each, as follows: (1) dry skin and mouth and heat vexation fever accompanied by uneasiness or restlessness for YnD and (2) and cold in the extremities up to the knees and elbows or beyond and diarrhea for YgD [35]. In summary, the QD, BD, YnD, and YgD scores might be beneficial for clarifying the characteristics of *deficiency* syndromes in clinical cases.

Convergent validity was assessed by investigating the relationships between the QBYY-Q scales and the CFS-K and FSS fatigue questionnaires. The convergent validity test showed that the QBYY-Q scales were significantly correlated with CFS-K and FSS scores (0.29 < *r* < 0.72).

Additionally, our study explored biochemical differences in the blood between the CF and control groups. The blood biochemistry and complete blood cell counts fell within the normal laboratory ranges [14, 35–37]. However, a CF diagnosis with YgD was significantly correlated with hemoglobin (*r* = −0.28) and red blood cell counts (*r* = −0.29), and a CF diagnosis with YnD was significantly correlated with chloride levels (*r* = 0.28). Statistically negative correlation coefficients of hematocrit (*r* = −0.37), hemoglobin (*r* = −0.35), red blood cells (*r* = −0.32), aspartate transaminase (*r* = −0.29), and alanine transaminase (*r* = −0.28) were observed in CF patients with BD. These data suggest that CF patients showed differences in physiological homeostasis.

YgD of the spleen and kidney is characterized by cold limbs, listlessness, cold and pain in the waist and knee joints, a pale tongue with a white coating, and a deep and thready pulse [9]. The cross-tabulation results of CF and the PIs showed that the YgD OR (4.68, CI 1.347–19.65) was significantly higher than the ORs of the other patterns (Table 6). The use of a questionnaire for *deficiency* syndromes might help to identify appropriate *qi*, *blood*, *yin*, and *yang* nourishing treatments for CF patients.

The YgD OR was significantly higher than the ORs of the other patterns (Table 7). However, the reliability of the YgD-Q (α = 0.717) was lower than that of the other patterns, indicating that some of the YgD-Q items might not have accurately reflected the YgD pattern. In other words, the QBYY-Q did not distinguish YgD from QD because YgD simultaneously included QD. The ORs suggested that the QBYY-Q scores might be beneficial for clarifying the characteristics of *deficiency* syndromes in clinical cases.

**Fig. 3:**
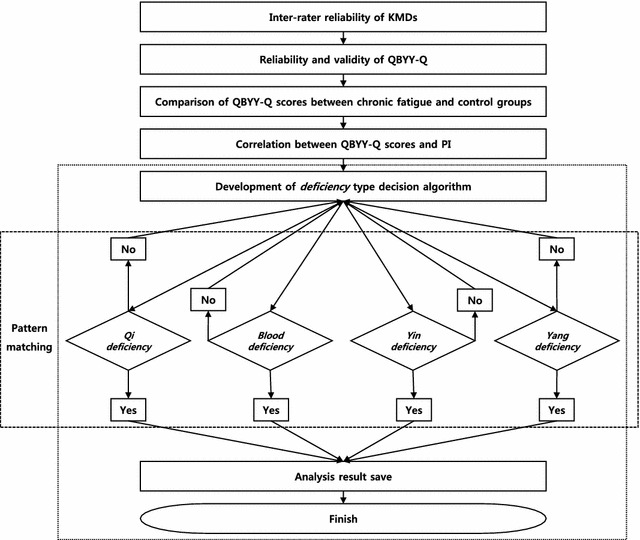
Development of an algorithm for *deficiency*-type identification

The ORs derived from the multinomial logistic regression analysis demonstrated that each QD, BD, YnD, and YgD-Q score was positively associated with each corresponding *deficiency* pattern (adjusted ORs: qi score = 0.041, blood score = 5.877, yin score = 12.57, yang score = 13.56, reference category: QD). We constructed an algorithm for estimating the PI of a CF patient, including data on age, sex, and QBYY-Q scores, and tested its predictability (Fig. 3). We developed and validated this algorithm based on the diagnostic results and QBYY-Q scores to predict the PIs of patients with CF. To evaluate the distinction possibility of the four *deficiency* patterns, we developed and validated an algorithm based on diagnostic results and QBYY-Q scores. The algorithm was developed and validated based on bootstrap resampling (B = 500) using ordinary multinomial logistic regression (precision = 0.608) and subscales of each *deficiency* pattern questionnaire, based on EFA (precision = 0.530) [38]. The selected final model depended on the simulation results. Bootstrap validation with 500 resamples of the selected model produced moderate predictive performance, with Cox & Snell *R*^2^ = 0.346, agreement = 0.523, κ = 0.369, area under the curve = 0.750, precision = 0.53, and recall = 0.535. The prevalence and severity of ICF and CFS varied according to ethnicity and psychosocial factors [39]. By validating the results, we confirmed the possibility of diagnosis using the QBYY-Q. Objective measurement of the subjective severity of fatigue is difficult, but it is essential in implementing therapy for unexplained CF [40, 41].

The study was limited by the small number of patients and the single-center design. Although CFS and ICF can be diagnosed using international guidelines, these standards are different from PI symptoms and signs, which are very difficult to standardize. However, we generated reference data that are applicable to future studies. Thus, the results of the present study could be used to improve the care of patients with CF and CF-related disorders and to facilitate research on anti-CF therapies. Large clinical trials in multiple centers on PI and the evaluation of the therapeutic effects on CF in randomized clinical trials are needed. Additional studies are required to assess the correlations between CF type and other demographic and clinical characteristics.

## Conclusion

The QBYY-Q was a suitable instrument for estimating the influences of *qi*, *blood*, *yin*, and *yang deficiencies* in CF.

## Additional files

10.1186/s13020-016-0092-y Approval number of Clinical Research Information Service (CRIS).
10.1186/s13020-016-0092-y Informed consent form.
10.1186/s13020-016-0092-y Complaints and symptoms of *Qi blood Yin Yang deficiency* questionnaire.

## Abbreviations

KM: Korean medicine
CFS: chronic fatigue syndrome
ICF: idiopathic chronic fatigue
CF: chronic fatigue
FSS: fatigue severity scale
CFS-K: Korean-translated Chalder fatigue scale
PSS: perceived stress scale
QBYY-Q: *qi blood yin yang deficiency* questionnaire
PI: pattern identification
KMD: Korean medical doctor
QD: *qi deficiency*
QD-Q: *qi deficiency* questionnaire
BD: *blood deficiency*
BD-Q: *blood deficiency* questionnaire
YnD: *yin deficiency*
YnD-Q: *yin deficiency* questionnaire
YgD: *yang deficiency*
YgD-Q: *yang deficiency* questionnaire
SBP: systolic blood pressure
DBP: diastolic blood pressure
KMO: Keiser Meyer Olkin
FL: factor loading
OR: odds ratio
