# Supplementary material for: Validation of the qi blood yin yang deficiency questionnaire on chronic fatigue
Source: Chin Med. 2016 May 2;11:24. doi: 10.1186/s13020-016-0092-y (PMC4852426; doi:10.1186/s13020-016-0092-y)
Supplement: Supplementary file 1 — 10.1186/s13020-016-0092-y Approval number of Clinical Research Information Service (CRIS). [file 13020_2016_92_MOESM1_ESM.pdf]

Background

Scientific Title	A Study on Reliability and Validity of the 'Qi Blood Yin Yang Deficiency Questionnaire'			
CRIS Registration No.	KCT0001199			
Investigate	No	Registered at Other Registry	No	

Contact Details & Status

Contact Person for Principal Investigator / Scientific Queries	Teakwon Ahn	Cheonan Korean Medicine Hospital of Daejeon University			
Study Center	Single	Participating Institute Name	Cheonan Korean Medicine Hospital of Daejeon University		
Overall Recruitment Status	Completed	Primary Completion Date	2014-06-30	Study Completion Date	2014-06-30
Target Sample Size	150	Date of First Enrollment	2014-05-12	State of First Enrollment	Actual

Source of Monetary/Material Support & Sponsor Organiztion

Source of Monetary /Material Support 1	Korea Institute of Oriental Medicine
Sponsor Organization 1	Korea Institute of Oriental Medicine

Study Summary

Lay Summary	This clinical study on chronic fatigue is investigative research that seeks to report the correlations between questionnaire results and syndrome differentiation diagnosis made by doctors, and to verify reliability and validity of 'Qi Blood Yin Yang Deficiency Questionnaire' which is completed as self -administered/reported questionnaire by both chronic fatigue group and non-chronic fatigue group (control).

Study Design

Study Type	Observational Study	Observational Study Model	Case-control	Time Perspective	Crosssectional
Cohort/Group Number	2				

Cohort/ Group 1	Cohort/ Group Label	Chronic fatigue group	Group Description	Subjects(n=120) diagnosed with chronic fatigue syndrome and Idiopathic Chronic Fatigue for using chronic fatigue syndrome criteria of Centers for Disease Control and Prevention(CDC). Compare and analyze 'Qi blood yin yang deficiency questionnaire score' of Chronic fatigue group and none chronic fatigue group
Cohort/ Group 2	Cohort/ Group Label	none chronic fatigue group	Group Description	Subjects(n=30) diagnosed with none chronic fatigue for using chronic fatigue syndrome criteria of Centers for Disease Control and Prevention(CDC). Compare and analyze 'Qi blood yin yang deficiency questionnaire score' of Chronic fatigue group and none chronic fatigue group
Biospecimen Retention		DNA Collect Archive Sample without DNA		
Biospecimen Description		blood, urine		

Subject Eligibility

Study Population Description	In this study, the standards for chronic fatigue syndrome and idiopathic chronic fatigue defined by Centers for Disease Control and Prevention (CDC) in 1994 are applied; 120 males and females aged over 20 and under 39 years who are diagnosed with chronic fatigue syndrome or idiopathic chronic fatigue, and 30 males and females aged over 20 and under 39 years who are not diagnosed with chronic fatigue syndrome or idiopathic chronic fatigue will be recruited.				
Sampling Method	non-probility sampling				
Condition(s) /Problem(s)	* Symptoms, signs and abnormal clinical and laboratory findings , NEC<br>F48.0 피로증후군<br>R53 권태감 및 피로			Rare Disease	No
Gender	Both	Age	20(Year) ~ 39(Year)	Accepting Healthy Volunteers	Yes
Inclusion Criteria	(1) males and females aged 20 to 39; (2) the presence of unexplained fatigue that is continuous and repetitive for more than 6 months; (3) not chronic fatigue syndrome and idiopathic chronic fatigue; (4) agreement of doctor diagnosis; and (5) those who consent to participate in this trial and sign an informed consent statement after listening to a clear explanation of the purpose and characteristics of this clinical trial				
Exclusion Criteria	(1) over 14 score of Perceived Stress Scale-10item(PSS-10item); (2) disagreement of 2 doctors' diagnoses; (3) the presence of the following conditions in the subject's past history that might trigger chronic fatigue: (a) organic causes, such as acute or chronic liver disease (for example, hepatitis, liver cirrhosis), anemia, tuberculosis, chronic lung disease, cardiovascular disease (for example, heart failure , hypertension), endocrine/metabolic disease (for example, diabetes, thyroid gland disease, severe obesity), autoimmune disease (for example, rheumatoid arthritis, systemic lupus erythematosus, multiple sclerosis), malignant tumors, or infectious disease; and (b) psycho-social causes, such as depression, anxiety neurosis, recent severe stress, schizophrenia, alcoholism, or an eating disorder (anorexia nervosa, bulimia nervosa); (4) subjects who have taken the following drugs within the past 2 weeks: antihypertensive drugs, antidepressants, anti-anxiety agents, hypnotics, or antihistamines; (5) pregnant or breast-feeding women; (6) subjects who are participating in other clinical trials; (7) subjects who are overworked; (8) subjects who have experienced a hypersensitive reaction after clinical laboratory test; (9) subjects who equipped to a cardiac pacemaker ; (10) subjects who do not provide informed consent; and (11) others whose clinical trial conductors are considered inappropriate for participating in this trial				

Outcome Measure(s)

Type of Primary Outcome	Not applicable			
Primary Outcome 1	Outcome	Questionnaire results on the syndrome differentiation of consumptive disease	Timepoint	After completion of the clinical trials
Secondary Outcome 1	Outcome	Korean medical doctors' diagnosis results on the syndrome differentiation	Timepoint	After completion of the clinical trials
Secondary Outcome 2	Outcome	Health-related clinical pathology examination results	Timepoint	After completion of the clinical trials
Secondary Outcome 3	Outcome	Score of stress response inventory (Fatigue Severity Scale, Chalder Fatigue Scale)	Timepoint	After completion of the clinical trials
Secondary Outcome 4	Outcome	Analytical variables of tongue image obtained through tongue imaging system	Timepoint	After completion of the clinical trials
Secondary Outcome 5	Outcome	Output variables of dermometer (DDFAO)	Timepoint	After completion of the clinical trials

Publication

Number of publications	0
