# Supplementary material for: Validation of the qi blood yin yang deficiency questionnaire on chronic fatigue
Source: Chin Med. 2016 May 2;11:24. doi: 10.1186/s13020-016-0092-y (PMC4852426; doi:10.1186/s13020-016-0092-y)
Supplement: Supplementary file 2 — 10.1186/s13020-016-0092-y Informed consent form. [file 13020_2016_92_MOESM2_ESM.pdf]

대전대학교 천안한방병원 vision 한방 Service

Ver.1.0

<Informed consent form>

Signature page

Title of the project

Clinical Trial for the Syndrome Differentiation of Consumptive Disease

ID: ME2-D97-H | | | |

The purpose of this project is to validate the PI questionnaires through clinical trials seeking to identify correlations between CF and responses to the *Qi Blood Yin Yang Deficiency* Questionnaire (QBYY-Q).

I have read and understood the information about the project. I have been given the opportunity to ask questions about the project and my participation. I understand I can withdraw at any time without giving reasons and that I will not be penalized for withdrawing nor will I be questioned on why I have withdrawn. The use of the data in research, publications, sharing and archiving has been explained to me. I agree to sign and date this informed consent form.

Participant’s Printed Name	Signature of Participant	Date Signed
Parent’s/Guardian’s Printed Name	Signature of Participant	Date Signed
Principal Investigator Printed Name	Signature of Participant	Date Signed

Attention

This project has been reviewed and approved by an Institutional Review Board of Cheonan Korean Medicine Hospital of Daejeon University.
