# Supplementary material for: Validation of the qi blood yin yang deficiency questionnaire on chronic fatigue
Source: Chin Med. 2016 May 2;11:24. doi: 10.1186/s13020-016-0092-y (PMC4852426; doi:10.1186/s13020-016-0092-y)
Supplement: Supplementary file 3 — 10.1186/s13020-016-0092-y Complaints and symptoms of Qi blood Yin Yang deficiency questionnaire. [file 13020_2016_92_MOESM3_ESM.pdf]

Complaints and symptoms of *Qi blood Yin Yang deficiency* questionnaire

Pattern identification	Item	Complaint	Symptom
Qi deficiency	QD-Q 01	I feel listless	Feel listless
	QD-Q 02	I feel too languid to work	Fatigue
	QD-Q 03	I feel feeling of helplessness	Helplessness
	QD-Q 04	I am attempting the impossible	Overwork
	QD-Q 05	I feel weak and cannot move around much	Weakness
	QD-Q 06	I haven't had an appetite lately	Loss of appetite
	QD-Q 07	I sweat often these days	Sweating
	QD-Q 08	My voice is getting weaker	Strained voice
	QD-Q 09	I have symptoms of proctoptosis and hysteroptosis	Proctoptosis and hysteroptosis
Blood deficiency	BD-Q 01	I feel giddy	Giddiness
	BD-Q 02	My heart keeps pounding	Heart pounding
	BD-Q 03	I often have cramps	Cramping
	BD-Q 04	I have a pale complexion	Pale complexion
	BD-Q 05	My hair is friable	Friable hair
	BD-Q 06	I have a bad complexion of lips, lids and nails	Bloodless lips, lids and nails
	BD-Q 07	I have a dry and tired eyes	Dry and tired eyes
	BD-Q 08	I frequently dream during sleep	Frequent dreaming
Yin deficiency	YnD-Q 01	I do not gain weight despite eating fully	Weight maintenance
	YnD-Q 02	I feel a dull pain in my lower back or knees	Dull pain in the lower back or knees
	YnD-Q 03	I feel heat deep in the body, hands and feet	Body steaming
	YnD-Q 04	I feel flushed in the afternoon	Afternoon flushing
	YnD-Q 05	I have rough skin	Rough skin
	YnD-Q 06	My eyesight is failing	Failing of eyesight
	YnD-Q 07	I have a dry mouth	Dry mouth
	YnD-Q 08	I have fevers in the afternoon	Afternoon fever
	YnD-Q 09	I sweat during sleep	Sweating during sleep
Yang deficiency	YgD-Q 01	I prefer warm things to cold things	Warm thing preference
	YgD-Q 02	I prefer warm beverages to cold beverages	Hot beverage preference
	YgD-Q 03	My hands and feet feel cold	Cold hands and feet
	YgD-Q 04	I urinate frequently during feel cold	Frequent urination
	YgD-Q 05	I have diarrhea in the morning	Diarrhea
	YgD-Q 06	I have soft stools	Soft stool
